# Do Cross-Group Differences in Life Satisfaction Reflect Measurement Bias or True Differences in Life Satisfaction? Evidence from a Dutch National Sample

**DOI:** 10.3390/ijerph191811687

**Published:** 2022-09-16

**Authors:** Mohsen Joshanloo

**Affiliations:** Department of Psychology, Keimyung University, 1095 Dalgubeol Boulevard, Dalseo-Gu, Daegu 42601, Korea; mjoshanloo@kmu.ac.kr

**Keywords:** satisfaction with life scale, measurement invariance, The Netherlands, LISS, life satisfaction

## Abstract

The purpose of this study was to examine measurement invariance of the Dutch version of the Satisfaction with Life Scale between groups based on gender, age, education, perceived difficulty of the survey, perceived clarity of the survey, and national background. A nationally representative Dutch sample was used (N = 5369). Multiple-groups confirmatory factor analysis was conducted to test measurement invariance. Full metric and scalar invariance were supported for all groups studied. These results indicate that the items of the scale are understood and answered similarly by all groups. Therefore, the 5 items of the Satisfaction with Life Scale measure the same construct in all groups. In other words, the differences in the life satisfaction scores are indicative of actual differences in life satisfaction rather than measurement artifacts and biases. This means that the levels of life satisfaction can be meaningfully compared between groups in The Netherlands.

## 1. Introduction

To measure overall life satisfaction, respondents are asked to rate their overall life satisfaction on a scale from very unsatisfactory to very satisfactory. Life satisfaction scales are now used in many research and applied fields in small and large surveys around the world [1]. Life satisfaction data are increasingly being used to inform policy decisions at the community and societal levels [2], which primarily includes comparing satisfaction scores over time or comparing different groups on their life satisfaction scores. The satisfaction with life scale (SWLS) [3] is among the most widely used general life satisfaction scales. Like any other measurement tool, the SWLS can be biased in certain contexts. It may be that different groups understand the items in the scale differently or respond systematically differently to the items. When this is the case, group differences in scale scores do not reflect actual differences in life satisfaction, but rather differences in the measurement properties of the scale between groups. In other words, unless we are sure that the scale functions in a completely similar way in all groups, the mean differences between groups cannot be interpreted as actual differences in life satisfaction.

Measurement invariance refers to the similarity of a measurement instrument’s psychometric properties between groups [4]. The main goal of an analysis of measurement invariance in different groups (e.g., women and men) is to determine whether the items of a measure have the same factor structure, the same units of measurement (factor loadings), and the same item means (intercepts). Only if all of these parameters are similar across groups can we claim that the scale measures the same construct across those groups. If invariance is established, we can conclude that the same test score corresponds to the same level of the construct, regardless of which group a test taker belongs to [5]. When measurement invariance is established, differences in means can be interpreted as true differences in the construct measured by the scale. However, a lack of measurement invariance makes such comparisons ambiguous, to say the least, because individuals from different groups may perceive certain words or phrases or scale items or even the entire scale differently because of linguistic differences or differences in group-specific assumptions and beliefs [6]. It cannot be assumed that the SWLS or any other well-being scale means the same thing in different groups unless measurement invariance is statistically supported. Unfortunately, most studies comparing means of life satisfaction between different groups (e.g., age, gender, education, and nationality) do not examine measurement invariance. Only a fraction of previous studies has examined the invariance of the SWLS, which has led to alarming conclusions. Emerson et al. [7] provided a review of these studies. The majority of studies have examined measurement invariance across age, gender, and national groups. The results show that more than half of the studies supported measurement invariance between genders, while invariance between age and national groups was not supported in many of the studies. Thus, it is important to routinely test the invariance of satisfaction scales before drawing conclusions about group differences in life satisfaction.

The SWLS has been used in many studies in The Netherlands, and its basic psychometric properties (e.g., convergent validity and reliability) have been found to be satisfactory [8,9]. However, to our knowledge, the measurement invariance of the SWLS has never been investigated in The Netherlands. Therefore, The Netherlands provides a new opportunity to investigate the invariance of the SWLS, which was the aim of the present study. In this study, a large Dutch sample was used to examine the invariance of the SWLS across multiple groups. In addition to age and gender groups, the study also examined the invariance of the scale between groups with different levels of education. People with different levels of education may have different understandings of their lives, different standards for evaluating their lives, different interpretations of the SWLS items, or they may have different familiarity with or ease of answering the survey questions. For example, people with different levels of education respond differently to instructional manipulation checks [10,11] or show different levels of inattention when answering surveys [12]. Research in other fields, such as human values [13], have found evidence of non-invariance across educational levels. A Spanish study examined the invariance of the SLWS across educational levels and found full invariance [14]. The present study used a larger sample from another country to re-examine the invariance of the SWLS across educational levels.

The dataset used in this study contains two questions that were asked of all participants at the end of the survey. These two questions ask participants to rate how difficult or clear the survey questions were. Their inclusion provides a unique opportunity to quantify the perceived difficulty and clarity of the survey, and to examine the invariance of the SWLS across groups formed based on different levels of perceived difficulty and clarity. Individuals who perceive the survey questions to be unclear or difficult may respond differently to the survey questions than individuals who perceive the questions to be simple and clear. The purpose of the present study was to investigate whether these two groups of participants responded systematically differently to the questions or whether perceived difficulty and clarity had an influence on responses to the SWLS items.

Finally, The Netherlands is home to many individuals from various national backgrounds. Might it be that people from various backgrounds understand and respond to psychological scales differently? The answer is yes. Studies with immigrants versus natives in various countries have found non-invariance, for example, in scales of self-regulated cognitive strategies [15] and unmet needs [16]. Other studies across ethnic groups in the same nation have found a lack of measurement invariance, for example, for scales of internalizing and externalizing behavior problems [17], self-concept [18], and world assumptions [19]. One study found evidence for measurement invariance of the SWLS in older Hispanics and Whites in the United States [20]. Invariance of the SWLS across groups with different backgrounds cannot be assumed and must be tested in each country; therefore, this study investigated this issue in The Netherlands.

In summary, this study aimed to examine the invariance of the Dutch version of the SWLS between groups based on gender, age, education, perceived difficulty of the survey, perceived clarity of the survey, and national background. The aim is to find out whether the same construct of life satisfaction is measured in all these groups (i.e., whether life satisfaction has the same meaning in all these groups) and whether the items of the scale function similarly across groups.

## 2. Methods

### 2.1. Participants

Data are from the 13th wave of the LISS (Longitudinal Internet Studies for the Social Sciences) panel project, collected in 2021. The panel is based on a probability sample of households drawn from the Statistics Netherlands population register. LISS participants complete online questionnaires each month, which take about 15 to 30 min and for which they are paid. The SWLS scale is part of the personality section of the LISS core study, which is repeated annually. More information about study procedures and data can be found at: https://www.lissdata.nl/about-panel (accessed on 1 June 2022). The sample of this study consists of 5369 individuals (female = 53.8%, M_age_ = 52.81, SD_age_ = 18.653).

### 2.2. Measure

The SWLS [3] measures general life satisfaction. The five items of the scale are “In most ways my life is close to my ideal”, “The conditions of my life are excellent”, “I am satisfied with my life”, “So far I have gotten the important things I want in life”, and “If I could live my life over, I would change almost nothing”. The items are rated on a 7-point scale ranging from *strongly disagree* (1) to *strongly agree* (7). The Cronbach’s alpha was 0.902 in this study. 

### 2.3. Grouping Variables

Age quartiles were used to obtain four age categories with relatively similar sample sizes: 16–37 years, 38–55 years, 56–68 years, and 69–103 years. To increase the sample size of the groups, the education categories were combined into three categories: (1) primary school and junior high school, (2) senior high school and junior college, and (3) college and university. For the difficulty question (“Was it difficult to answer the questions?”), there were 5 response options (from 1 = *certainly not* to 5 = *certainly yes*). Two groups were formed to distinguish about 62% of respondents who chose 1 and 2 (not difficult) and about 38% who chose between 3 and 5 (difficult). For the clarity question (“Were the questions sufficiently clear?”), there were 5 response options (1 = *certainly not* to 5 = *certainly yes*). Two groups were formed to distinguish about 20% of respondents who chose between 1 and 3 (not clear) and about 80% who chose between 4 and 5 (clear). Two background groups were formed: those who indicated a Dutch background and those who indicated a first- and second-generation foreign background. Descriptive statistics for all groups are presented in Table 1, along with mean scores for life satisfaction.

## 3. Results

### 3.1. Factor Structure and Reliability

**Figure 1:**
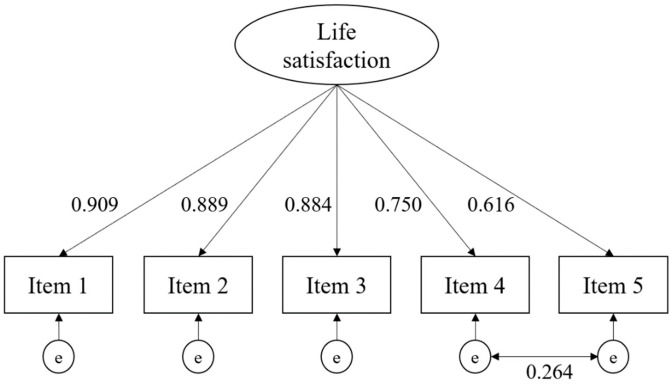
Modified CFA model. Note: e = error. Numbers are standardized coefficients.

Using the whole sample, a confirmatory factor analysis was conducted with the five items as indicators of a single factor of life satisfaction. The fit of this model was not excellent (Table 2, Model 1). The modification indices suggested that an item residual (error) covariance between items 4 and 5 would improve the fit. These two items are focused on the past while the first three items are focused on the present [21], and hence this modification is theoretically justifiable. In many other countries, this covariance has been found to improve model fit, including Norway, Serbia, and Germany [22,23,24]. A modified model (Model 2) was tested which provided an excellent fit to the data. This covariance was kept in all invariance models of the study. Standardized factor loadings for items 1–5 ranged between 0.616 and 0.909. This model is depicted in Figure 1. Model-implied omega reliability was 0.901 (10,000-replications; bias-corrected bootstrap confidence interval = 0.895–0.906), suggesting a high level of reliability.

### 3.2. Measurement Invariance

Under a multiple-groups confirmatory factor analysis framework, this study tested two types of invariance: metric and scalar invariance [4,25]. Metric invariance means that factor loadings are the same across groups. In other words, metric invariance indicates that each item contributes to the latent construct of life satisfaction to a similar extent across groups. Scalar invariance means that item intercepts are the same across groups (in addition to factor loadings). That is, participants who have the same score on the latent construct of life satisfaction have the same item scores regardless of which group they belong to. When scalar invariance is supported, we conclude that individuals with the same overall life satisfaction scores do not tend to answer the items of the SWLS systematically higher or lower in the groups studied. Before testing metric and scalar invariance, a configural invariance model is tested which serves as a baseline model. The configural invariance model is a confirmatory multigroup factor analysis model with no equality constraints on factor loadings or item intercepts. When this model fits the data well, it means that the same items measure our construct in all groups. Then, metric and scalar models are tested one at a time. Equality constraints are imposed on factor loadings to test for metric invariance, and while retaining factor loading constraints, equality constraints are imposed on the intercepts to test for scalar invariance. Criteria used for invariance were: ΔCFI ≥ −0.010, ΔRMSEA ≥ 0.015, and ΔSRMR ≥ 0.030 for metric invariance, and ΔCFI ≥ −0.010, ΔRMSEA ≥ 0.015, and ΔSRMR ≥ 0.010 for scalar invariance [26]. Models were tested using Mplus, with full information and robust maximum likelihood (MLR). 

The results of the tests for measurement invariance are shown in Table 2. As can be seen, none of the model fit deteriorations exceed the thresholds, and thus, full scalar invariance for the SWLS is supported across all groups tested.

## 4. Discussion

The results demonstrate configural invariance as well as full metric and scalar invariance across all groups in this Dutch sample. In this study, configural invariance means that: (1) one latent variable in all groups is sufficient to capture the common variance in the five items, and (2) a single latent variable of life satisfaction is measured with the same items in all groups. This essentially means that life satisfaction has the same basic factor structure in all groups studied here. However, configural invariance does not mean that individuals respond to the items in the same way in all groups. Metric invariance means that each item contributes to the latent construct of life satisfaction to a similar degree in all groups. In other words, the observed item differences are indicative of similar cross-group differences in the underlying construct of life satisfaction. Thus, the scores of an item that exhibits metric invariance can be meaningfully compared across groups. Finally, scalar invariance implies that cross-group differences in item means are due to differences in the means of the latent variable of life satisfaction. When an item is scalar invariant, it means that the item’s scores are not biased upward or downward between groups. Thus, individuals with the same level of the latent variable of life satisfaction have similar observed responses to the item regardless of which group they belong to [25,27]. Overall, full scalar invariance implies that the same construct is measured in all groups and that life satisfaction scores in The Netherlands can be compared across groups. Therefore, we are not comparing apples and oranges in research with the SWLS in The Netherlands.

Although full invariance of the SWLS for age groups has not been supported in some countries [7], full invariance across four age groups was supported in this study. This study is one of the first to examine measurement invariance of the SWLS across national backgrounds within a single country and across groups formed on the basis of self-reported survey difficulty and clarity, and thus we hope that the results contribute to the current discourse on the measurement of well-being and the measurement invariance literature in general.

In conclusion, these results suggest that, at least in The Netherlands, we can assume a common construct of life satisfaction across all groups, measured by the five items of the Dutch SWLS. The differences observed in the latent scores of life satisfaction are indicative of real differences in life satisfaction rather than measurement artifacts and biases. This is good news for research and policy related to mental well-being. Governments in many countries (including The Netherlands) have shown increasing interest in promoting mental well-being as a policy goal [28]. Initiatives to measure well-being in the general population are an important aspect of this new agenda. Accordingly, measures of life satisfaction are widely used in national and international surveys [29]. Therefore, it is critical to ensure that these measures are not biased against certain groups in society. This study showed that the SWLS provides a fairly unbiased assessment of life satisfaction in different groups of Dutch society and can certainly be used as a basis for policy decisions in this country.

## Figures and Tables

**Table 1:** Descriptive statistics for groups.

Grouping Variable	Group	N	M	SD
Gender	Male	2474	5.029	1.132
	Female	2891	5.050	1.144
Age quartile	1 (16–37)	1337	4.947	1.148
	2 (38–55)	1354	5.011	1.123
	3 (56–68)	1374	5.039	1.193
	4 (69–103)	1300	5.167	1.072
Education	Primary school and junior high school	1383	4.933	1.206
	Senior high school and junior college	1868	4.970	1.148
	College and university	2098	5.174	1.069
Difficulty	Not difficult (1–2)	3280	5.150	1.121
	Difficult (3–5)	2029	4.860	1.144
Clarity	Not clear (1–3)	1028	4.784	1.185
	Clear (4–5)	4281	5.100	1.118
Background	Dutch background	4237	5.100	1.102
	First- or second-generation foreign background	987	4.796	1.259

Means and SD are for the total SWLS score.

**Table 2:** Fit indices.

Model		X^2^	df	*p*	RMSEA	RMSEA 90% CI	CFI	SRMR	ΔRMSEA	ΔCFI	ΔSRMR
CFA models											
1	Original CFA model	271.792	5	0.000	0.100	0.090 0.110	0.969	0.029	-	-	-
2	Modified CFA model	92.753	4	0.000	0.064	0.053 0.076	0.990	0.013	-	-	-
Invariance-gender											
3	Configural	97.051	8	0.000	0.064	0.053 0.076	0.990	0.013	-	-	-
4	Metric	109.981	12	0.000	0.055	0.046 0.065	0.989	0.022	−0.009	−0.001	0.009
5	Scalar	135.095	16	0.000	0.053	0.045 0.061	0.987	0.026	−0.002	−0.002	0.004
Invariance-age											
6	Configural	100.756	16	0.000	0.063	0.051 0.075	0.991	0.013	-	-	-
7	Metric	125.406	28	0.000	0.051	0.042 0.060	0.989	0.036	−0.012	−0.002	0.023
8	Scalar	194.205	40	0.000	0.054	0.046 0.061	0.983	0.045	0.003	−0.006	0.009
Invariance-education											
9	Configural	93.919	12	0.000	0.062	0.051 0.074	0.991	0.013	-	-	-
10	Metric	109.547	20	0.000	0.050	0.041 0.060	0.990	0.022	−0.012	−0.001	0.009
11	Scalar	157.147	28	0.000	0.051	0.043 0.059	0.985	0.026	0.001	−0.005	0.004
Invariance-difficulty											
12	Configural	95.041	8	0.000	0.064	0.053 0.076	0.990	0.013	-	-	-
13	Metric	121.696	12	0.000	0.059	0.049 0.068	0.987	0.038	−0.005	−0.003	0.025
14	Scalar	140.415	16	0.000	0.054	0.046 0.063	0.986	0.038	−0.005	−0.001	0.000
Invariance-clarity											
15	Configural	97.784	8	0.000	0.065	0.054 0.077	0.989	0.013	-	-	-
16	Metric	109.623	12	0.000	0.055	0.046 0.065	0.988	0.018	−0.010	−0.001	0.005
17	Scalar	132.714	16	0.000	0.052	0.044 0.061	0.986	0.021	−0.003	−0.002	0.003
Invariance-background											
18	Configural	97.042	8	0.000	0.065	0.054 0.077	0.989	0.013	-	-	-
19	Metric	115.782	12	0.000	0.058	0.048 0.067	0.988	0.025	−0.007	−0.001	0.012
20	Partial metric	133.470	16	0.000	0.053	0.045 0.062	0.986	0.026	−0.005	−0.002	0.001

CI = confidence interval.

## Data Availability

The data and study materials are publicly available at https://www.lissdata.nl/access-data (accessed on 1 June 2022).
